# National Policy Influences of Contraceptive Prevalence and Method Mix Strategy: A Longitudinal Analysis of 59 Low- and Middle-Income Countries, 2010–2021

**DOI:** 10.9745/GHSP-D-23-00352

**Published:** 2024-04-29

**Authors:** Michael A. Cohen, Suzanne Gold, Arthur Ostrega, Mark Zingbagba

**Affiliations:** a Chemonics International, New York, NY, USA.; b Chemonics International, Washington, DC, USA.; c Chemonics International, Kumasi, Ghana.

## Abstract

Understanding the impact of family planning policy and actions is essential for building effective strategies to increase contraceptive use. This study identifies policies that correlate with modern contraceptive prevalence rate (mCPR) and private-sector contraceptive method mix strategies (the number of contraceptive methods offered in the private sector) in low-income and middle-income countries. While education, contraceptive choices, and economic growth are known determinants of contraceptive prevalence, many national policies intended to increase contraceptive prevalence in the short term to medium term have ambiguous evidence that they indeed do so. By developing beta and Poisson regression models using 12 years of reported Contraceptive Security Indicators Survey data (2010–2021) from 59 countries, this study investigated the effect of 20 independent variables on mCPR or method mix strategies. Furthermore, to help interpret the potential consequences of economic status, separate models segmented by gross national income (low, low-middle, and upper-middle) were assessed. Of 20 independent variables, 10 are implicated with mCPR and 6 with a method mix strategy. Of these, increasing the share of domestic financing (versus donor funding) for contraceptives had the broadest and strongest contribution. mCPR is also predicted by the existence of national insurance systems that cover contraceptive costs, contraceptive security committees, family planning logistics management information systems, and, inversely, by client fees. A comprehensive private-sector method mix strategy—which itself influences mCPR—is also driven by these, as well as the inclusion of more contraceptives on the national essential medicines list. These findings have implications for countries seeking to expand access to and use of contraceptives through policy initiatives.

## Abstract (precis)

Evidence from over a decade of Contraceptive Security Indicators survey data across 59 countries reveals a subset of finance, governance, and logistics policies that boost modern contraceptive prevalence rate and method-mix strategy.

## Teaser

**Key Findings:**

Based on an analysis of a dataset incorporating evidence from the Contraceptive Security Indicators survey (2010–2021):

- Increased national government contraceptive financing (in contrast to donor funding) is strongly correlated with modern contraceptive prevalence rate (mCPR) and private-sector contraceptive method mix strategy.
- Health insurance coverage for family planning is correlated with mCPR in middle- but not low-income countries.
- A logistics management information system that includes family planning commodities is strongly correlated with mCPR in low- and middle-income countries.

**Key Implications:**

- Ministries of health and international funding agencies should prioritize the policies and activities that show substantiated evidence of success in raising contraceptive prevalence.
- Stakeholders should acknowledge the benefits of financing contraceptives directly by national governments (versus external contributors) when allocating resources; however, funding agencies should continue to minimize any residual gaps in this fundamental investment to the extent possible.
- Health insurance coverage for family planning, while correlated with mCPR, may be related to decreased contraceptive choices in the private sector. To better capitalize on their investments, countries should recognize this paradox and devote efforts to encourage private-sector choice offerings.

## INTRODUCTION

Meaningful progress toward diverse national goals is often dependent on the achievement of reproductive health and family planning (FP) objectives. Improved FP outcomes contribute to the United Nations Sustainable Development Goals, including gender equality, health and well-being, and education. Thus, inquiries intended to increase the effectiveness of FP programming are valuable investments. Despite important gains in many countries toward national goals of increasing the modern contraceptive prevalence rate (mCPR), the United Nations Population Fund reports that an estimated 164 million women in low- and middle-income countries (LMICs) who want to avoid pregnancy are not currently using an effective contraceptive method.^1^ While several of the demographic and behavioral predictors of mCPR have been identified, including education and age,^2^ there remain gaps in evidence supporting the effectiveness of some policy strategies. Stronger evidence would help governments and international organizations advocate for and finance an array of approaches to strengthen FP programs to attain the health and socioeconomic benefits anticipated from increased contraceptive use. Better understanding of the relationships between FP-related policies and their intended outcomes is a fundamental step toward establishing efficient country-led strategies that lead to effective approaches, stronger systems, and a healthier population.

Better understanding of the relationships between FP-related policies and their intended outcomes is a fundamental step toward establishing efficient country-led strategies that lead to effective approaches, stronger systems, and a healthier population.

An essential component of effective FP is contraceptive security (CS), defined as the ability to reliably choose, obtain, and use quality contraceptives.^3^ Between 2010 and 2021, the CS Indicators Survey, developed by the U.S. Agency for International Development (USAID), was conducted in 63 countries. The survey assessed a broad range of potential CS levers, including measures of contraceptive financing; the number of methods offered; national policies, coordination, and leadership; and supply chain benchmarks.

This study focuses on policies and strategies intended to increase access to contraceptives rather than demand for contraceptives created by, for example, socioeconomic attributes of the population. In this analysis, we investigated 20 potential catalysts of CS (plus 2 known contextual variables: education and economy) in 59 LMICs to identify which correlate with mCPR and private-sector contraceptive method mix strategy (MMS). We define mCPR as contraceptive prevalence among women of reproductive age (15–49 years) who are married or in a union using at least 1 contraceptive method and is directly correlated with reductions in unintended pregnancy and maternal and child mortality.^4^^,^^5^ MMS is defined as the number of contraceptive methods that are included in a policy and/or were distributed or ordered by at least 1 private-sector entity within the previous 12 months.

When more contraceptive methods are available, mCPR increases.^6^^–^^8^ Therefore, the advantages of upstream policies and activities that strengthen strategies to expand method mix should be better understood. Based on the models with mCPR as the dependent variable, we identify private-sector outlets (not public sector, which includes government-funded and managed facilities, products, and services) correlated with mCPR. For this reason, we investigated potential predictors of contraceptive MMS in the private sector. This work addresses the policies driving the strategy as opposed to the availability of private-sector contraceptive methods. The approach taken here is intended to test the outcome of a coordinated national strategy designed to facilitate and support contraceptive use and identify elements associated with either mCPR or a strategy of expanded method mix in the private sector.

## METHODS

### Description of Data

The study relies on CS policy and strategy data captured by the CS Indicators Survey.^9^ Using a mixed-methods approach of qualitative and quantitative elements, the survey collected information from key stakeholders and primary source documents on factors that are expected to contribute to the availability and accessibility of affordable, quality-assured contraceptives. Spanning a period from 2010 to 2021, the survey has been conducted in 63 LMICs purposively selected based on past and present USAID priority FP countries, membership in the Ouagadougou Partnership, or having made an FP2020/FP2030 commitment. Not every LMIC is represented, and not every country included was assessed each year (Table 1 and Supplement Table S1 for survey response data by selected years). Data about national-level CS policies was collected from ministries of health to inform 254 variables. Comprehensive descriptions of all variables and archived data are available online,^10^ and Table 2 includes definitions of variables identified as contributing explanatory variables in any model. This analysis employs “all-country” models that assess data on 59 countries in which the CS Indicators Survey was conducted, as well as models that segment countries into low-income and combined lower-middle and upper-middle income groupings according to World Bank classifications using the gross national income (GNI) (Atlas method).^11^

**TABLE 1.** All 59 Low- and Middle-Income Countries Included in Analyses of Contraceptive Security Indicator Survey Data on mCPR^a^^,^^b^

**Country**	**World Bank Region**	**2021 World Bank Classification by Income Status**	**mCPR**								
			**2010**	**2011**	**2012**	**2013**	**2014**	**2015**	**2017**	**2019**	**2021**
Afghanistan	SA	low	16	15.9	15.7	16.3	17.3	18.3	20.0	21.4	23.2
Albania	ECA	upper-middle	10.3	9.1	*8.0*	7.0	*6.1*	*5.3*	*4.0*	*4.3*	*4.9*
Angola	SSA	low-middle	*11.8*	*12.1*	*12.3*	*12.5*	*12.6*	*12.8*	13.4	14.2	15.2
Argentina	LAC	upper-middle	*65.6*	*65.5*	*65.6*	*66.0*	*66.2*	*66.4*	*66.5*	67.5	*67.4*
Armenia	ECA	upper-middle	30.0	29.4	29.6	30.6	32.4	33.6	30.0	*29.9*	*30.5*
Azerbaijan	ECA	upper-middle	15.0	15.2	15.8	16.5	*27.4*	*27.7*	*28.7*	*29.9*	*31.1*
Bangladesh	SA	low-middle	51.0	52.2	53.2	54.0	54.2	54.3	53.4	53.8	54.5
Benin	SSA	low-middle	*7.4*	*7.8*	*8.2*	8.9	9.7	10.5	11.8	13.0	14.4
Bolivia	LAC	low-middle	*37.9*	39.1	40.2	*41.5*	*42.7*	*43.90*	*45.8*	47.2	*48.7*
Burkina Faso	SSA	low	15.1	16.2	17.6	19.1	20.8	22.6	26.7	29.2	30.6
Burundi	SSA	low	*18.6*	*20.6*	22.8	23.3	23.3	23.1	23.4	25.2	27.4
Cameroon	SSA	low-middle	*14.4*	*14.6*	*15.0*	*15.5*	15.7	15.7	15.4	15.6	16.6
Cape Verde	SSA	low-middle	*56.5*	*56.5*	*56.4*	*56.2*	56.2	56.0	55.6	55.5	56.2
Côte d’Ivoire	SSA	low-middle	*11.9*	*12.3*	*12.7*	*13.5*	*14.3*	15.6	18.0	20.1	*20.3*
Dominican Republic	LAC	upper-middle	47.3	47.7	46.1	47.7	44.3	45.2	42.2	41.9	*65.3*
DRC	SSA	low	*6.6*	6.9	7.3	*7.5*	8.3	9.3	11.8	13.8	15.2
Ecuador	LAC	upper-middle	*67.2*	*68.1*	*68.9*	*69*	*69*	*69.5*	*70.7*	73.0	*72.3*
El Salvador	LAC	low-middle	65.6	66	66.4	66.7	67.1	67.3	67.8	68.2	68.6
Ethiopia	SSA	low	25.1	27.8	30.5	33.1	35.2	36.0	36.4	38.1	40.5
Gambia	SSA	low	*9.4*	9.1	9.0	*9.1*	*9.8*	*10.7*	*13.2*	*15.8*	*17.8*
Georgia	ECA	upper-middle	28.3	29	29.5	29.9	30.2	30.5	*31.3*	*32.1*	*33.1*
Ghana	SSA	low-middle	18.6	18.6	18.6	18.6	18.6	18.6	18.6	18.6	18.6
Guatemala	LAC	upper-middle	44.8	45.8	46.9	47.8	48.7	49.5	50.9	52.1	53.2
Guinea	SSA	low	*5.2*	*5.2*	5.3	5.7	6.4	7.1	9.0	10.8	12.1
Haiti	LAC	low	*29.3*	30.2	30.9	31.2	31.4	31.6	32.1	33.3	34.7
Honduras	LAC	low-middle	62.0	63.2	64.2	64.8	65.3	65.6	66.2	66.8	67.3
India	SA	low-middle	48.1	48.0	47.9	47.8	47.7	47.8	48.4	49.3	50.3
Indonesia	EAP	low-middle	*58.9*	*59.4*	*59.7*	60.0	*59.9*	59.5	*58.5*	*58.9*	*59.6*
Kenya	SSA	low-middle	44.4	47.4	50.4	53.3	56.3	59.1	59.8	58.1	58.2
Kyrgyz Republic	ECA	low-middle	*36.3*	*35.2*	*34.5*	*34.7*	*35.3*	*35.7*	*36.7*	37.8	38.9
Lao PDR	EAP	low-middle	*41.3*	*42.5*	*43.9*	*45.2*	*46.5*	*47.8*	*50.1*	51.7	53.0
Liberia	SSA	low	14.3	15.7	17.2	18.8	20.2	21.6	*23.3*	24.4	25.5
Madagascar	SSA	low	30.9	32.2	33.4	34.7	35.9	37.1	39.6	42	44.2
Malawi	SSA	low	42.6	45.7	48.9	51.9	54.7	57.0	59.8	61.7	63.1
Mali	SSA	low	9.0	9.6	10.3	11.1	12.2	13.3	15.4	17.2	18.8
Mauritania	SSA	low-middle	*9.4*	*9.9*	*10.3*	12.4	13.3	14.2	*11.5*	*11.4*	11.8
Mexico	LAC	upper-middle	*67.6*	*67.8*	*67.9*	*68*	*68.1*	*68.3*	*68.9*	69.6	*69.9*
Mozambique	SSA	low	12.5	12.5	12.5	12.5	12.5	12.5	12.5	12.5	12.5
Nepal	SA	low-middle	43.8	43.6	43.8	43.9	44.2	44.1	44.4	45.5	46.7
Nicaragua	LAC	low-middle	75.6	76.5	77.1	77.4	77.7	77.9	*78.2*	78.5	*78.7*
Niger	SSA	low	*11.2*	*11.7*	*12.1*	*12.6*	13.2	13.2	13.2	13.2	13.2
Nigeria	SSA	low-middle	10	10.1	10.1	9.9	10.3	10.8	11.7	13.1	14.6
Pakistan	SA	low-middle	23.8	24.6	25.3	25.8	25.7	25.6	25.3	26.4	28.0
Paraguay	LAC	upper-middle	65.5	65.7	65.8	65.8	65.9	66.0	*66.3*	66.7	*67.2*
Peru	LAC	upper-middle	*51.1*	*51.9*	*52.2*	*51.7*	52.1	53.0	*54.3*	55.7	57.0
Philippines	EAP	low-middle	35.4	36.2	37.1	37.9	38.5	39.0	40.1	41.2	42.3
Rwanda	SSA	low	40.9	44.2	45.7	46.4	46.8	47.2	49.1	51.1	53.1
Senegal	SSA	low-middle	12.2	13.3	15.1	17.2	19.4	21.3	25.0	25.8	27.3
Sierra Leone	SSA	low	*9.7*	*11.4*	*13.2*	15.2	16.6	17.8	*20.2*	21.8	23.6
South Africa	SSA	upper-middle	*59.0*	*58.3*	*57.6*	*56.9*	56.1	55.6	*55.5*	*56.2*	*57.2*
South Sudan	SSA	low	*4.8*	5.3	5.4	5.4	5.3	5.3	*5.7*	6.3	6.9
Tanzania	SSA	low-middle	27.4	28.4	29.3	30.1	31.0	31.9	34.0	36.3	38.7
Togo	SSA	low	*14.6*	*15.2*	15.9	16.8	17.6	18.5	20.5	22.2	23.9
Uganda	SSA	low	23.8	25.1	26.1	27.1	28.5	30.9	34.9	37.8	40.5
Ukraine	ECA	low-middle	49.2	49.7	50.1	50.7	51.1	51.6	52.5	*53.4*	54.5
Vietnam	EAP	low-middle	*67.1*	*66.9*	*66.4*	*66.0*	*65.6*	*65.6*	*65.7*	65.4	*65.2*
Yemen	MENA	low	22.0	22.9	23.9	25.0	25.8	26.6	*28.4*	*30.0*	31.7
Zambia	SSA	low-middle	38.6	40.5	42.2	43.8	44.9	45.6	46.7	47.9	49.5
Zimbabwe	SSA	low-middle	58.0	58.8	60.4	62.3	64.1	65.6	67.0	67.7	68.3
Average mCPR			35.3	33.3	33.1	32.5	34.6	35.9	34.3	39.3	37.2
Average mCPR (low income)			22.9	21.3	21.0	23.2	22.7	23.6	27.0	26.8	28.5
Average mCPR (middle income)			41.2	40.5	42.6	39.2	42.7	44.0	39.1	46.6	43.6
Population composition											
Geographic distribution	EAP: n=4, 6%	ECA: n=7, 11%	LAC: n=13, 21%		MENA: n=1, 2%		SA: n=6, 10%		SSA: n=32, 51%		
Income distribution	Low: n=19, 30%		Low-middle: n=29, 46%		Upper-middle: n=14, 22%		High: n=1, 2%				

Abbreviations: DRC, Democratic Republic of the Congo; ECA, European and Central Asia; EAP, East Asia and Pacific; LAC, Latin America and the Caribbean; mCPR, modern contraceptive prevalence rate; MENA, Middle East and North Africa; PDR, People’s Democratic Republic; SA, South Asia; SSA, sub-Saharan Africa.

^a^ mCPR data for women aged 15–49 years, married or in a union, estimates and projections compiled by United Nations Population Fund.

^b^ mCPR data in italics indicates the Contraceptive Security Indicators Survey was not implemented in the country for that year (country/year excluded from models).

**TABLE 2.** Definitions^a^ of Family Planning Indicators Identified as Contributing Explanatory Variables in Any Model

**Policy/Practice**	**Definition**	**Variable Type**
FP2020 commitment	Whether a country made a commitment to the FamilyPlanning2020 partnership (before 2020). This indicator has been collected since 2017.	Binary
National government share of spending on contraceptives	Percentage of total government and donor spending on contraceptives in the previous year attributable to national government spending on contraceptives in the same period. Spending is normally measured through the value of commodities delivered during the 12-month period in question, although in some cases it is measured through commodities purchased or shipped during the period. Collected since 2010.	Percentage
Existence of an LMIS that includes FP/RH commodities	The existence of a national LMIS that collects data on contraceptive commodities. Collected since 2017.	Binary
FP commodities subject to duties in the private sector	Whether FP commodities procured by the private sector are subject to import duties. Collected since 2010.	Binary
CS committee meeting frequency	A CS committee is defined as a national committee with a mandate to address contraceptive commodity security. It must have some aspect of CS as part of its terms of reference. Committee roles range from advisory to decision-making. Meeting frequency includes both remote and in-person meetings; response options include: 0, “a few times a year” (1–3 times), and “many times a year” (4 or more times). Collected since 2010.	Ordinal
Count of contraceptive methods on the NEML	The number of contraceptive methods included on a country’s NEML. Ranges from 0–10 as defined by the WHO’s Model Essential Medicines List^11^ plus the contraceptive patch and fertility-awareness based methods. Collected since 2010.	Continuous
Method mix strategy (private sector)	The number of FP methods (0–13) that are offered through the private sector. A product is considered offered even if not currently in stock, if ordered or distributed within the previous 12 months, and/or that is required to be offered according to national guidelines, protocols, and laws. Collected since 2010.	Continuous
Method mix strategy (public sector)	The number of FP methods (0–13) that are offered through the public sector. A product is considered offered even if not currently in stock, if ordered or distributed within the previous 12 months, and/or that is required to be offered according to national guidelines, protocols, and laws. Collected since 2010.	Continuous
Fees charged to clients for FP commodities in the public sector	Assesses whether clients at public health facilities are charged fees for FP/RH commodities by policy. Collected since 2010.	Binary
Family planning charges covered by health insurance	Whether a public, government, or other national health insurance scheme covers the cost of FP services or commodities for public-sector clients (not applicable if no fees are charged). Collected since 2017.	Binary
School enrollment, primary and secondary (gross), GPI (ratio)	Ratio of girls to boys enrolled at primary and secondary levels in public and private schools as reported by UNESCO Institute for Statistics.	Continuous
GDP per capita (current US$)	GDP divided by midyear population. GDP is the sum of gross value added by all resident producers in the economy plus any product taxes and minus any subsidies not included in the value of the products. It is calculated without making deductions for depreciation of fabricated assets or for depletion and degradation of natural resources. Data are in current U.S. dollars. World Bank and OECD.	Continuous

Abbreviations: CS, contraceptive security; FP, family planning; GDP, gross domestic product; GPI, gender parity index; LMIS, logistics management information system; NEML, national essential medicines list; RH, reproductive health; UNESCO, United Nations Educational, Scientific and Cultural Organization.

^a^ For definitions of the full set of 20 indicators included in models are included in Supplement Table S2.

For each country grouping, 2 dependent variables were tested: mCPR^12^ and private-sector MMS. Contraceptive prevalence data was compiled by the United Nations Population Division from multiple household surveys; where data were unavailable, the Family Planning Estimation Tool was used to estimate values.^13^ With strong evidence linking wealth and school enrollment to contraceptive use,^14^^,^^15^ the all-country mCPR (mCPR-All) and MMS (MMS-All) models incorporate per capita gross domestic product (GDP) and school enrollment (gross enrollment ratio, primary and secondary gender parity index) as context variables.^16^^,^^17^ To avoid multicollinearity, GDP was excluded from models segmented by income.

### Data Preparation

A reconciled historical CS dataset was compiled (www.ghsupplychain.org), from which we selected relevant policy variables based on the results of a correlation matrix and were in sync with domain expertise. Where the Pearson correlation between 2 variables was greater than or equal to a threshold of 60%, 1 was dropped to reduce the problem of multicollinearity. Additionally, numerical values were assigned for categorical or ordinal survey responses, and detailed categories were collapsed into baskets. Where possible, responses were validated by triangulating source documents, comparing against prior data, and assessing outliers against current contexts. Table 3 summarizes the available data and that which was ultimately incorporated in the regression models presented here.

**TABLE 3.** Overview of the Harmonized CS Indicators Survey Dataset

**Original (From Harmonized CS Indicators Survey Dataset)**	**Post-Cleaning**	**Comment**
254 variables	20 selected	Driven by the correlation matrix. A threshold of 60% triggered a requirement to use domain knowledge to choose between highly correlated variables.
63 countries	59 countries selected	Three countries—Botswana, Russia, and Sri Lanka— were removed because of insufficient context variable data. Chile was removed because it is classified as a high-income country.
9 context variables	2 selected	GDP per capita (current US$) School enrollment: primary and secondary (gross), GPI; ratio

Abbreviations: GDP, gross domestic product; GPI, gender parity index.

### Dependent Variables

mCPR is defined as the proportion of women aged 15–49 years, married or in a union, who are using at least 1 modern contraceptive. MMS is defined as the number of methods offered in the private sector (defined as ordered or distributed by a private-sector entity within the previous 12 months or required to be offered according to national guidelines, protocols, or laws).

### Modeling

We assessed policies by examining how they predict mCPR and MMS. For each dependent variable, we developed analytical models for all countries and, to explore contextual dependencies, low-income and middle-income countries. Regression models were selected by identifying the relationships between dependent and independent variables based on dependent variable distribution (Table 4).

**TABLE 4.** Profile of Regression Models

**Model**	**Dependent Variable**	**Model Type**	**RMSE (normalized RMSE)**
mCPR-All	mCPR, all 59 countries modeled	Mixed-effects beta regression	0.127 (0.002)
mCPR-LI	mCPR, LI countries (GNI<=US$1046)	Mixed-effects beta regression	0.078 (0.134)
mCPR-MI	mCPR, MI countries includes lower-MI (GNI US$1046–US$4095) and upper-MI (GNI US$4096–US$12695) countries	Mixed-effects beta regression	0.149 (0.205)
MMS-All	Number of private-sector methods offered for all 59 countries modeled	Poisson regression	2.142 (0.163)
MMS-LI	Number of private-sector methods offered, LI countries	Poisson regression	2.006 (0.154)
MMS-MI	Number of private-sector methods offered, lower-MI and upper-MI countries	Poisson regression	2.063 (0.159)

Abbreviations: GNI, gross national income; mCPR, modern contraceptive prevalence rate; LI, low income; MI, middle income; MMS, method mix strategy; RMSE, root mean square error.

### Evaluation

We assessed the magnitude and direction of the regression coefficient of each variable, incorporating domain knowledge and the existing literature (when available) to support results. We accounted for multiple measures within the same country by re-estimating models using a mixed effects approach for fitting generalized linear mixed models using the beta regression family. The potential correlation of the error terms for observations from the same country was assessed using the Breusch-Pagan test for heteroskedasticity. This test examines whether the variance of the errors from a regression model is dependent on the predicted values, helping to identify heteroscedasticity patterns where the spread of residuals may change across different levels of predicted values. To confirm the absence of overdispersion, we conducted a sensitivity analysis using linear regression models. To ensure the effectiveness in mirroring data appropriately in the Poisson models, comparison with a negative binomial model was used to demonstrate the level of dispersion across multiple parameters by comparing the dispersion parameters Akaike’s Information Criterion and Bayesian Information Criterion. An assessment of multicollinearity, constant variance of the error terms, and the coefficient of determination were also incorporated.

### Data Types

In the final models, 20 variables were retained: 13 binary, 2 ordinal, 3 continuous, and 2 percentage. All 3 continuous variables are bounded (between 1 and 12 and between 1 and 13), although treated as continuous to facilitate analysis. For ordinal data, a distinction is made between levels of “yes” responses to capture frequency and quality of intervention. Table 2 defines the variables identified as correlates in any model; the full definitions of the 20 variables included in models are presented in Supplement Table S2.

mCPR is a percentage; therefore, we use beta regression, which estimates the correlation between the independent variables and the logit of the dependent variable, as shown in the following formula.

logit(mCPR) = log(mCPR/(1-mCPR)) = α + βX

where X is independent variables, α is intercept, β is model estimates, exp is exponent, the constant term and estimates of the independent variables.

Exponentiating and taking the multiplicative inverse of both sides:

(1-mCPR)/mCPR = 1/exp(α + βX)

Partialling out the fraction of the left-side of the equation and adding 1 to both sides:

1/mCPR = 1 + (1/(exp(α + βX))

We can obtain the probability of a change in mCPR from this equation by changing 1 to the common denominator and taking the multiplicative inverse again, as shown in the following equation.

mCPR = exp(α + βX)/(1+ exp(α + βX))

For this study, the independent variables (X) are policy indicators whose change may be associated with a change in the mCPR, quantified by β for each independent variable. Α estimates the change in mCPR without the effect of any policy variable. The interpretation of β depends on the type of data that constitutes the independent variable (X).

To model the predictors of the number of methods offered in the private sector, we use a Poisson model because the independent variable counts the number of “yes” responses to each of 13 potential contraceptive methods offered through the country’s private sector. Using cluster-robust standard errors provided consistent estimates and addressed the issue of heteroscedasticity.

## RESULTS

### Predictors of Modern Contraceptive Prevalence Rate

Of 20 CS policies included in the all-country model (mCPR-All) after validation and cleaning, 6 correlated with mCPR (Table 5).

**TABLE 5.** Contribution to Dependent Variables of All Indicators, by Model

	**mCPR**						**FP Private-Sector MMS**					
	mCPR-All		mCPR-LI		mCPR-MI		MMS-All		MMS-LI		MMS-MI	
Model name	0.127 (0.002)		0.078 (0.134)		0.149 (0.205)		2.142 (0.163)		2.006 (0.154)		2.063 (0.159)	
RMSE (normalized RMSE)	***β***	*P* Value	***β***	*P* Value	***β***	*P* Value	***β***	*P* Value	***β***	*P* Value	***β***	*P* Value
Independent variables outcomes^a^												
Financing related												
Government budget line exists for contraceptives	−.480	.323	−.469	.202	.484	.580	−.022	.502	−.019	.742	−.031	.570
Total expenditures as a percent of forecast	.515	.206	.513	.543	.518	.212	−.024	.129	.060	.243	−.031	.355
National government share of contraceptive spending	**.685**	**.000** ^b^	**.588**	**.012** ^c^	**.710**	**.000** ^b^	**.120**	**.007** ^d^	**.1869**	**.037** ^c^	**.1469**	**.036** ^c^
Duties on commodities, private sector	−.474	.380	*−.437*	*.033* ^d^	−.484	.739	.033	.492	−.042	.540	.109	.241
Duties on commodities, public sector	.508	.732	.557	.051	−.479	.524	.045	.143	.049	.405	.052	.420
Partnership with GFF	.524	.241	.523	.352	−.556	.051	−.003	.945	.084	.070	−.063	.369
Client charges, services, public sector	*−.450*	*.032* ^c^	−.476	.465	−.447	.160	.051	.273	**.163**	**.017** ^c^	.032	.694
Client charges, commodities, public sector	−.460	.109	*−.432*	*.038* ^c^	−.454	.193	.027	.551	−.055	.430	.027	.708
FP charges covered by health insurance	**.610**	**.012** ^c^	.539	.360	**.637**	**.034** ^c^	*−.253*	*.001* ^b^	*−.343*	*.001* ^b^	−.163	.177
Governance related												
CS Committee has legal status	.520	.256	**.547**	**.029** ^c^	.512	.646	−.027	.309	.047	.339	−.006	.907
CS strategy established	−.471	.352	−.468	.603	−.456	.192	.031	.526	−.108	.217	.075	.395
CS Committee: meeting frequency	**.529**	**.009** ^d^	**.536**	**.024** ^c^	.523	.107	**.043**	**.006** ^d^	**.087**	**.015** ^c^	.012	.672
Contraceptive MMS, private sector	**.514**	**.000** ^b^	**.510**	**.006** ^d^	**.513**	**.037** ^c^						
Contraceptive MMS, public sector	.504	.473	−.488	.160	.503	.646						
FP providers trained in implant and IUD insertion and removal, public sector, %	.513	.233	.508	.511	.514	.319	−.007	.686	−.020	.580	.002	.945
FP2020 Commitment established by country	−.473	.159	**.564**	**.002** ^d^	.473	.426	**.073**	**.015** ^c^	−.022	.662	.105	.053
Count of contraceptive commodities on NEML	.507	.172	−.499	.947	.508	.171	**.020**	**.001** ^b^	.004	.771	**.022**	**.039** ^c^
Logistics related												
LMIS includes FP commodities	**.551**	**.015** ^c^	**.984**	**.000** ^b^	**.970**	**.000** ^b^	.059	.150	.079	.172	.020	.773
Context/control												
School enrollment, primary and secondary (gross), GPI (ratio)	**.970**	**.000** ^b^	**1.000**	**.000** ^b^	**1.000**	**.000** ^b^						
GDP per capita, current US$	**.5**	**.000** ^b^					**.061**	**.006** ^d^				

Abbreviations: CS, contraceptive security; FP, family planning; GDP, gross domestic product; GFF, Global Financing Facility; GPI, gender parity index; IUD, intrauterine device; LI, low income; LMIS, logistics management information system; mCPR, modern contraceptive prevalence rate; MI, middle income; MMS, method mix strategy; NEML, national essential medicines list; RMSE, root mean square error.

^a^ Independent variables with *P*-values less than .05 were considered to be significant contributors to mCPR. Of those independent variables with *P*<.05, higher β values indicate a greater effect on the dependent variable (mCPR or private sector MMS). Values in bold have a positive correlation and values in italics have a negative correlation.

^b^ *P*<.001.

^c^ *P*<.05.

^d^ *P*<.01.

**FIGURE 1:**
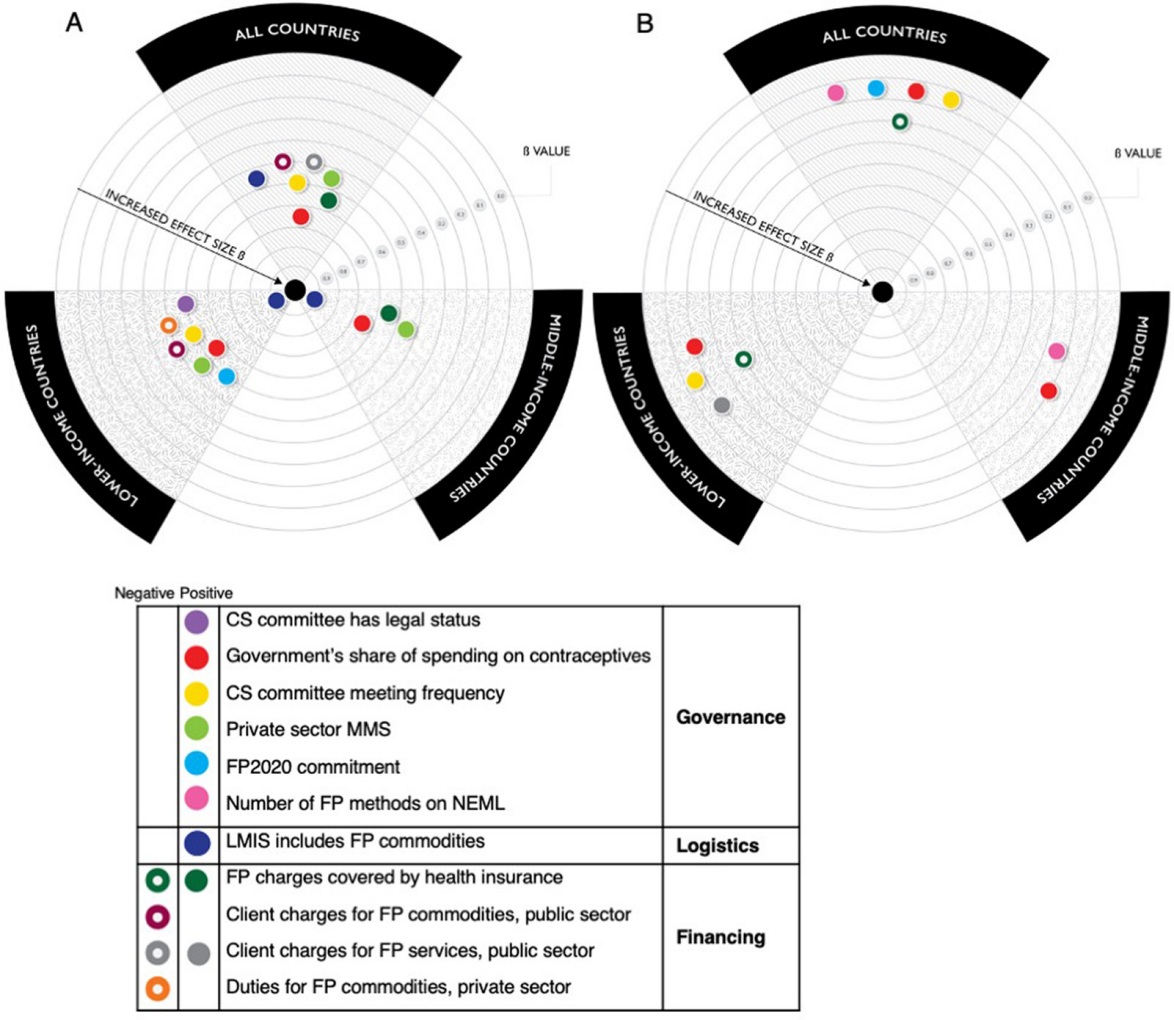
Independent Variables Contributing Significantly^a^ to the Dependent Variable for (A) mCPR Models, and (B) Method Mix Strategy Models Abbreviations: CS, contraceptive security; FP, family planning; LMIS, logistics management information system; mCPR, modern contraceptive prevalence rate; MMS, method mix strategy; NEML, national essential medicines list. ^a^ *P*≤.05. Dots closer to the center have higher β values.

In the all-country model (mCPR-All; root mean squared error [RMSE]=0.127), significantly correlated financing-related variables were the national government’s share of spending on contraceptives (β=.685) and the presence of a national insurance scheme that covers FP charges (β=.610). Charging fees to clients for FP services in the public sector was negatively correlated (β=−.450). Governance-related dependent variables significantly correlated with mCPR across all countries were CS committee meeting frequency (β=.528) and the number of contraceptive methods offered in the private sector (β=.514). Having a logistics management information system (LMIS) that includes FP commodities (β=.551) was positively correlated. A summary of predictors for each dependent variable, by model and by economic segmentation of countries is presented in Table 5 and Figure 1.

Significantly correlated financing-related variables were the national government’s share of spending on contraceptives and the presence of a national insurance scheme that covers FP charges.

**FIGURE 2:**
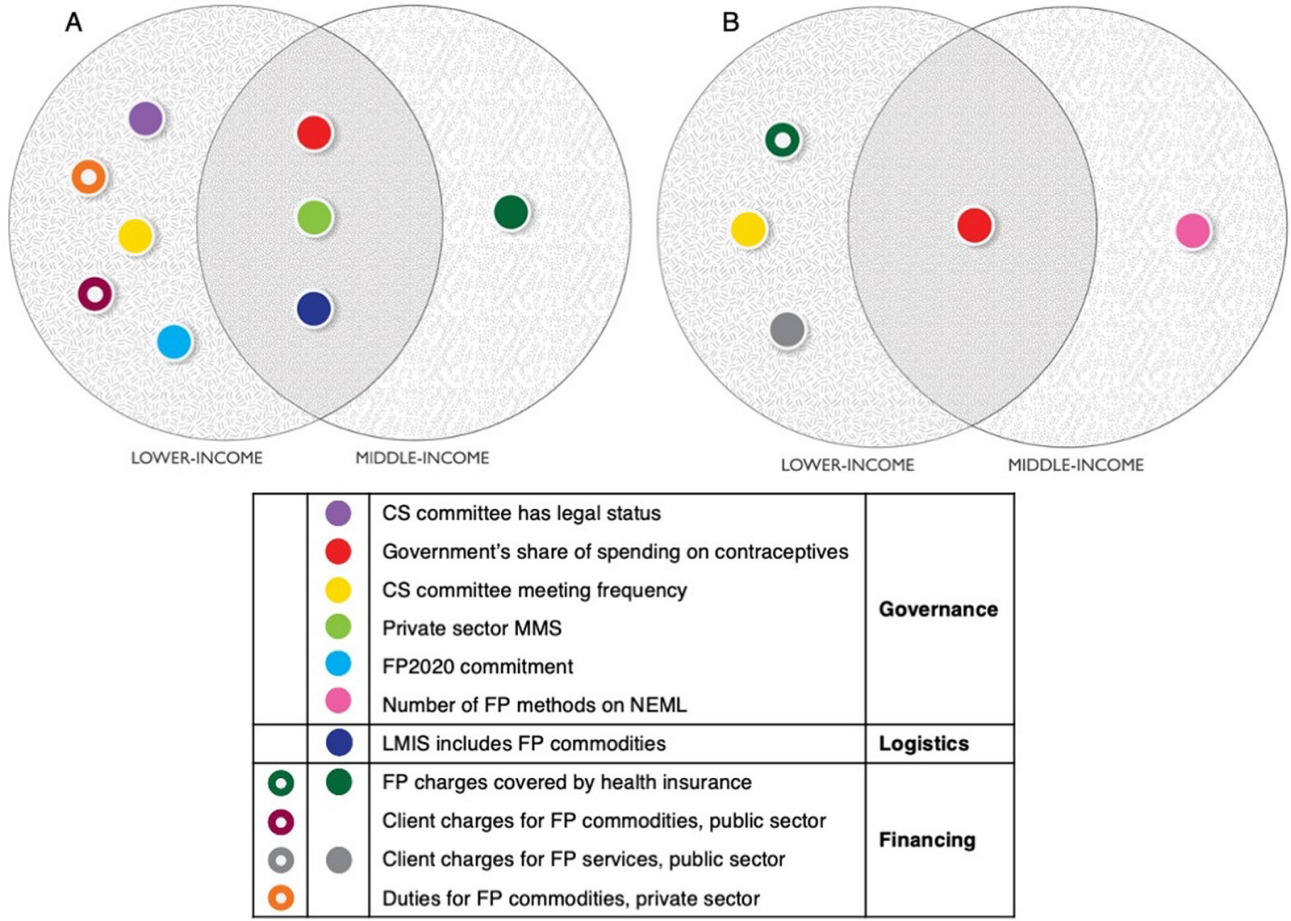
Comparison of Independent Variables Contributing Significantly^a^ in Low-Income and Middle-Income Countries to (A) mCPR Models, and (B) Private Sector Method Mix Strategy Models Abbreviations: CS, contraceptive security; FP, family planning; LMIS, logistics management information system; mCPR, modern contraceptive prevalence rate; MMS, method mix strategy; NEML, national essential medicines list. ^a^ *P*≤.05. Some dependent variables behave differently across different economic contexts.

Segmenting by World Bank GNI classification levels revealed variations between low-income and middle-income countries (Figure 2). The low-income model (mCPR-LI) identified 8 correlates of mCPR. Positive correlates of mCPR for low-income countries were national government’s share of spending on contraceptives (β=.588), CS committee meeting frequency (β=.536), CS committee having legal status (β=.547), number of contraceptive methods offered in the private sector (β=.510), making an FP2020 commitment (β=.564), and having an LMIS that includes FP commodities (β=.984). Negative correlates of mCPR for low-income countries were private-sector duties on contraceptives (β=−.437) and charging fees to clients for FP commodities (public sector) (β= −.433).

The middle-income model (mCPR-MI) identified 4 positive correlates of mCPR: national government’s share of spending on contraceptives (β=.710), FP charges covered by health insurance (β=.637), number of contraceptive methods offered in the private sector (β=.513), and having an LMIS that includes FP commodities (β=.970).

### Correlates of Private-Sector Method Mix Strategy

MMS is manipulated by policy levers that national governments use to influence the approach to increasing the number of contraceptives on offer rather than on the physical availability of contraceptives. Reflecting the influence of the private, but not public sector, in these models, the dependent variable in each of the 3 related MMS models (MMS-All, MMS-LI, and MMS-MI) is the number of contraceptive method types providers are expected to offer, as defined above.

Analysis of all countries identified 5 policies that are correlated with private-sector MMS (Table 5). In this model (MMS-All, RMSE=2.041), national government’s share of spending on contraceptives (β=.120), CS committee meeting frequency (β=.043), having an FP2020 commitment (β=.073), and the count of contraceptive commodities on the NEML (β=.020) were positively correlated with MMS. FP charges covered by health insurance was negatively correlated (β=−.253).

In low-income countries (MMS-LI, RMSE=1.917), the national government’s share of spending on contraceptives (β=.187), charging fees to clients for FP services (public sector, β=.163), and CS committee meeting frequency (β=.087) were positively correlated with MMS in the private sector. FP charges covered by health insurance were negatively correlated in the MMS-LI model (β=−.343). In the middle-income country model (MMS-MI, RMSE=2.012), the national government’s share of spending on contraceptives (β=.147) and the count of contraceptive commodities on the NEML (β=.022) helped drive MMS.

## DISCUSSION

Of the 20 policies investigated here, 10 correlated with mCPR, and 6 with an expanded private-sector MMS (Table 5 and Figure 1).

### Governance Related

Through constitutional powers and resource mobilization, national governments use diverse mechanisms to influence social and economic development. To advance contraceptive availability, strategic objectives are frequently launched that include the establishment of policies that are intended to support a wider method mix offering, product registration and licensing through a legislative framework, procurement of contraceptives, and delivery to health facilities. A comprehensive strategy of improving and maintaining related health outcomes requires the coordinated action of diverse stakeholders, along with robust accountability and coordination mechanisms supported by national-level CS committees. They coordinate stakeholders’ political and technical roles and demonstrate a country’s commitment to FP. In practice, CS committees range from an advisory body that produces recommendations to one with formal decision-making and implementation responsibilities. Committee participation typically includes public, private, nongovernmental, and faith-based organizations. Private-sector participants, whose influence may be direct and coordinated, was 33% in 2021.^9^ CS committees are often expected to coordinate national-level commodity forecasting and drive distribution system strengthening, providing them with influence to coordinate available contraceptives through the development of government interventions.^18^ Committees also advance MMSs by supporting the formal registration of diverse contraceptive methods.

Although middle-income country models do not associate CS committees with mCPR, success may be masked by the integration of committee functions into related government units.^9^ CS committee roles are complex, fluid, and susceptible to global and local political and funding priorities.

These models’ results predict private- but not public-sector influence of MMS on mCPR, but we speculate that the influence of method availability in the public sector was masked by additional obstacles faced by public providers that affect the supply chain, including funding, staffing, and bureaucratic complexities.

Despite the robust correlation between private-sector MMS and mCPR, when contemplating the introduction of additional methods, bear in mind the complexities inherent in real-world environments. Approaches intended to expand contraceptive choice vary from country to country, such as whether there is an official policy that governs the offering of a method or the support of messages promoting acceptance and use of unfamiliar methods. The extent to which policies and procurements translate into availability on pharmacy shelves may also vary by geography and timing. For example, Haakenstad et al. considered the additional costs of introducing new contraceptive methods (such as training and additional supply chain requirements) versus improving the availability and accessibility of widely accepted existing methods.^19^

The extent to which policies and procurements translate into availability on pharmacy shelves also may vary by geography and timing.

If mCPR in the population is increased by offering a robust mix of contraceptive methods in the private sector, as demonstrated repeatedly by others, could CS policies also be driving MMS? The policy environment that decision-makers work within must support a strategy that establishes a favorable governance structure and encourages choice. Our models recognized 2 such components that drive private-sector MMS: the number of contraceptive methods on an NEML and having an FP2020 commitment. An NEML is, at minimum, a compendium of medicine needs for a basic health care system. Inclusion of products on the list typically triggers activities to promote the registration, procurement, distribution, and associated education efforts that are required to ensure availability.

As with CS committees, an FP2020 commitment signals a country’s intent to support CS by pledging to take steps to accelerate progress toward expanding FP access. With a formal commitment to FP2020 goals, progress toward expanding contraceptive access is made in many, but not all, countries and is reflected by the correlation with mCPR in low-income countries.

### Financing Related

Financial support of public health, including insurance strategies, is intended to foster the use of the health system by eliminating fees as a barrier to accessing FP services. Insurance coverage of FP charges directly addresses clients’ burden of FP costs and was positively correlated with mCPR in all and middle-income countries. Similarly, fees charged to clients for FP services (in all countries) or commodities (in low-income countries) in the public sector were negatively correlated with mCPR. The negative association of user fees with contraceptive prevalence is further supported by Tiendrebeogo et al.^20^ who reported an 86% increase (versus control) in the likelihood of using contraception by eliminating user fees in 8 districts of Burkina Faso.

The argument for instituting user fees may be rooted in a desire to provide improved services to the client, and we do observe a weak association between fees and private-sector MMS in low-income countries. However, the FP programming is ultimately aimed at reducing unmet need, and in the “all-country” model, the fees are negatively correlated with contraceptive prevalence.

Low-income countries enacting duties on imported FP commodities in the private sector, potentially indirectly increasing client costs, are negatively correlated with mCPR. Considering the long-term economic benefits generated by FP, short-term losses of tariffs may be fiscally justifiable and should be factored into country strategies as investment approaches.

The influence of cost on usage remains elusive. Korachais et al. reported that 3 of 4 studies reviewed suggested that demand for contraception is not sensitive to cost or user fees.^21^ Health insurance coverage has been linked to increased access to and use of health services in Rwanda^22^ and Colombia.^23^ However, insurance coverage was not associated with contraceptive use in a study of 20 African countries.^24^ Insurance coverage of FP is complex: before a recent push to include FP in insurance and health benefits packages, these schemes tended to prioritize urgent curative services over preventive care, excluding FP. Furthermore, many governments already fully or partially subsidize FP, especially in low-income countries, negating the need for their inclusion in insurance coverage and potentially explaining why it does not appear as a correlate in the mCPR-LI model.

Another measure of a government’s commitment to contraceptive procurement is the share of spending the country shoulders. The national governments’ share of contraceptive spending was correlated strongly with mCPR across all models. The percent of costs borne by a country in FP, or any health initiative, go beyond aggregating available resources.^25^ Asymmetries inherent in the donor-recipient relationship may obscure advantageous strategies and ultimately impede the achievement of desired goals. Ownership and commitment cannot be bought but are achieved through a consultative process that encourages the commitment of investments for health goals that are accurately targeted to reflect the country’s priorities at a point in time. Contraceptive prevalence requires more than the procurement of contraceptives; the choices governments make indicate both a pledge and a responsibility to ensure success by reinforcing those investments with policies and infrastructure needed to realize their goals. The apparent success of self-funding may be driven by this combination of increased resource availability and country ownership.

In all and low-income countries, the support for contraceptives through an insurance scheme was negatively correlated with MMS. While a national policy that provides insurance coverage for contraceptives was associated with mCPR, it appeared to do so despite, and possibly at the expense of, an expanded MMS in the private sector. Effects on fertility as a result of high-quality FP programs (for example, as defined by a “public-sector FP program impact score”) have been well established.^26^ Here, we focus on a unique attribute of national programs: government support of contraceptive costs. While compelling evidence justifies national insurance schemes that include support for contraceptives, considering the influence that method choice also has on contraceptive prevalence, efforts should be made to ensure that expanded options are incorporated into health insurance practices to take full advantage of the investments.

Although our results link specific benefits of government investment to mCPR and MMS, the existing reality is entangled with other influences. Corruption, non-health-related infrastructure inadequacies, and ineffective health policies may offset government spending on health and lead to minimal or negative effects on health outcomes. However, overall, evidence suggests government spending likely contributes to positive health outcomes.^27^^–^^29^ There are links between government spending on health and decreased infant mortality in LMICs^30^ and maternal mortality in sub-Saharan Africa.^31^ In fact, although the total expenditures as a percent of forecast were not correlated with mCPR, that the government’s own share of this expenditure is so strongly associated with both mCPR and MMS in every model assessed suggests that raising political capital may be as influential as monetary and requires a different but coordinated approach.

### Logistics Related

Supply-side factors related to contraceptive access are fundamental components of CS. One of the most influential variables in the mCPR models is the existence of an LMIS that collects data on FP commodities. This allows for more integrated and streamlined contraceptive supply processes, promoting greater access, lower discontinuation rate and unmet need, and mCPR. Unreliable access is often cited as a reason for nonuse or discontinuation of contraception.^32^ An LMIS that collects data on FP commodities enables the active management of contraceptive commodity stock levels. An effective LMIS can also engender client trust and confidence in a health care system.^33^ Current trends of investment in digital infrastructure create an opportunity to fund, develop, and implement an LMIS with a contraceptive component.

One of the most influential variables in the mCPR models is the existence of an LMIS that collects data on FP commodities, allowing for streamlined contraceptive supply processes and greater access.

We note that the lack of correlation between having a national LMIS (which is typically designed, installed, and implemented by ministries of health) and contraceptive MMS in the private sector was unsurprising. These systems are designed for and implemented by the public sector. Although there are known examples of private-sector facilities incorporated into it, these are isolated and atypical.

### Limitations

CS Indicators Survey data are self-reported through a process facilitated by key staff at government ministries of health, donor agencies, nongovernmental organizations, and private-sector entities. Through the years, multiple respondents from a single country have provided answers to the survey questions. Although we confirm data where possible by triangulating sources, this is not possible for all indicators. For example, we cannot document government spending on contraceptives with source records but rely on trend, outlier analysis, and published data in evaluating results. The survey was conducted by 2 USAID projects (USAID DELIVER PROJECT: 2010–2015; USAID Global Health Supply Chain Program-Procurement and Supply Management project: 2017–2023), and question wording has evolved; responses were harmonized to allow comparability. A time lag is expected between policy implementation and anticipated outcome; analysis cannot consistently capture the date of initial policy implementation. There may also be discrepancies between policy reporting and implementation. For example, the measure of duties levied in a country identifies a policy in place but does not assess the scope or amount of duties collected.

In policy analyses, LMICs are frequently bundled together, although this may obscure differences between the 2 populations that could be insightful when devising and promoting strategies. In fact, we find there are distinct differences between the groups (Figure 2). GNI per capita is a blunt tool that obscures disparity, motivation, and other potential underlying factors, making it difficult to establish the root causes of difference.

Dependent variables that our analysis found to be not significant in their impact on mCPR or private-sector MMS may contribute in other ways to the sustainability and equity of FP activities.

## CONCLUSION

This study advances several policies that are correlated with mCPR and MMS, 2 objectives of FP programming. By supporting policies that improve the availability, options, and regulation of contraceptives, governments and donors can affect outcomes to promote effective FP strategies.

Demand-driven contraceptive use is contingent on socioeconomic factors, entrenched beliefs, and societal norms surrounding fertility and gender that may take decades to change. A population’s education and national economies typically require many years to transform. In contrast, the policy environment is more flexible, and the application of these tools in the right context to improve contraceptive method offerings and diversify the supply of contraceptives is an important strategy to consider.

## Supplementary Material

GHSP-D-23-00352-supplement.pdf

## Notes

**Peer Reviewed**

**First Published Online:** April 12, 2024.
