# Supplementary material for: National Policy Influences of Contraceptive Prevalence and Method Mix Strategy: A Longitudinal Analysis of 59 Low- and Middle-Income Countries, 2010–2021
Source: Glob Health Sci Pract. 2024 Apr 29;12(2):e2300352. doi: 10.9745/GHSP-D-23-00352 (PMC11057802; doi:10.9745/GHSP-D-23-00352)
Supplement: GHSP-D-23-00352-supplement.pdf [file GHSP-D-23-00352-supplement.pdf]

Supplement to: Cohen MA, Gold S, Ostrega A, Zingbagba M. National policy influences of contraceptive prevalence and method mix strategy: a longitudinal analysis of 59 low- and middle-income countries, 2010–2021. *Glob Health Sci Pract*. 2024;12(2):e2300352. https://doi.org/10.9745/GHSP-D-23-00352

Supplement Table S1. Definitions of all indicators included in models

		2012			2014			2017			2019			2021		
		All	Low Income	Middle Income	All	Low Income	Middle Income	All	Low Income	Middle Income	All	Low Income	Middle Income	All	Low Income	Middle Income
**Government's share of spending on contraceptives (%)**		n= 35	n=15	n= 20	n= 42	n= 17	n= 25	n= 34	n= 15	n= 19	n= 47	n= 17	n= 30	n= 40	n= 18	n= 22
	**Top quintile**	7	0	7	10	0	10	6	0	6	14	0	14	6	0	6
	**Second quintile**	4	0	4	1	1	0	2	1	1	1	0	1	5	1	4
	**Third quintile**	1	0	1	5	2	3	3	0	3	6	2	4	4	2	2
	**Fourth quintile**	3	2	1	6	2	4	6	3	3	5	1	4	4	1	3
	**Bottom quintile**	20	13	7	20	12	8	17	11	6	21	14	7	21	14	7
**Fees charged to clients for FP commodities**		20%	22%	18%	22%	21%	23%	31%	40%	38%	29%	28%	29%	23%	16%	29%
		n= 40	n= 18	n= 22	n= 45	n= 19	n= 26	n= 36	n= 15	n= 21	n= 49	n= 18	n= 31	n= 43	n= 19	n= 24
**FP charges covered by health insurance**		N/A	N/A	N/A	N/A	N/A	N/A	29%	40%	22%	53%	50%	55%	25%	22%	27%
		N/A	N/A	N/A	N/A	N/A	N/A	n= 14	n= 5	n= 9	n= 17	n= 6	n= 11	n= 24	n= 9	n= 15
**Existence of LMIS with FP commodities**		N/A	N/A	N/A	N/A	N/A	N/A	N/A	N/A	N/A	92%	94%	90%	93%	95%	92%
		N/A	N/A	N/A	N/A	N/A	N/A	N/A	N/A	N/A	n= 49	n= 18	n= 31	n= 43	n= 19	n= 24
**CS committee meeting frequency*** (0-2 scale)		1.3	1.4	1.3	1.5	1.4	1.5	1.7	1.6	1.7	1.4	1.5	1.4	1.4	1.4	1.3
		n= 38	n= 17	n= 21	n= 40	n= 18	n= 22	n= 34	n= 15	n= 19	n= 45	n= 18	n= 27	n= 40	n= 19	n= 21
**Commercial sector methods offered**** (average #, 0-13)		8	7	9	8	8	9	8	8	8	9	8	9	9	8	10
		n= 40	n= 17	n= 23	n= 46	n= 19	n= 27	n= 36	n= 15	n= 21	n= 47	n= 16	n= 31	n= 42	n= 18	n= 24
**SDP stockout severity** (>25%)		47%	53%	43%	58%	59%	57%	25%	25%	25%	41%	46%	36%	19%	13%	27%
		n= 38	n= 17	n= 21	n= 40	n= 17	n= 23	n= 20	n= 8	n= 12	n= 27	n= 13	n= 14	n= 31	n= 16	n= 15
**Client charges for FP commodities**		20%	22%	18%	22%	21%	23%	31%	0.2	38%	29%	28%	29%	23%	16%	29%
		n= 40	n= 18	n= 22	n= 45	n= 19	n= 26	n= 36	n= 15	n= 21	n= 49	n= 18	n= 31	n= 43	n= 19	n= 24
**Intermediate supplier delivery**		N/A	N/A	N/A	N/A	N/A	N/A	N/A	N/A	N/A	32%	20%	38%	33%	25%	39%
		N/A	N/A	N/A	N/A	N/A	N/A	N/A	N/A	N/A	n= 44	n= 15	n= 29	n= 39	n= 16	n= 23
**Commercial sector import duties**		66%	61%	70%	60%	59%	62%	34%	27%	40%	55%	39%	66%	34%	26%	41%
		n= 38	n= 18	n= 20	n= 43	n= 17	n= 26	n= 35	n= 15	n= 20	n= 47	n= 18	n= 29	n= 41	n= 19	n= 22

*Average frequency from 0-2, where 0 is never, 1 is a few (1 or 2-3 in 2019 and 2021, 1-2 prior to 2019), and 2 is many (4 or more in 2019, 2021, 3-5 or 6 or more in 2017 and prior).

**Until 2017, the maximum number of methods included in the survey was 11. Beginning in 2017, it was 13.

Supplement to: Cohen MA, Gold S, Ostrega A, Zingbagba M. National policy influences of contraceptive prevalence and method mix strategy: a longitudinal analysis of 59 low- and middle-income countries, 2010–2021. *Glob Health Sci Pract*. 2024;12(2):e2300352. https://doi.org/10.9745/GHSP-D-23-00352

Supplement Table S2. Definitions of all indicators included in models

**Policy/Practice**	**Definition**	**Variable Type**
FP2020 commitment	Whether a country made a commitment to the FamilyPlanning2020 partnership (prior to 2020). This indicator has been collected since 2017.	Binary
National government share of spending on contraceptives	Percentage of total government and donor spending on contraceptives in the previous year attributable to national government spending on contraceptives in the same period. Spending is normally measured through the value of commodities delivered during the 12-month period in question, although in some cases it is measured through commodities purchased or shipped during the period. Collected since 2010.	Percentage
Existence of an LMIS that includes FP/reproductive health (RH) commodities	The existence of a national logistics management information system (LMIS) that collects data on contraceptive commodities. Collected since 2017.	Binary
FP commodities subject to duties in the private sector	Whether FP commodities procured by the private sector are subject to import duties. Collected since 2010.	Binary
CS committee meeting frequency	A CS committee is defined as a national committee with a mandate to address contraceptive commodity security. It must have some aspect of CS as part of its terms of reference. Committee roles range from advisory to decision-making. Meeting frequency includes both remote and in-person meetings; response options include: 0, "a few times a year" (1-3 times), and "many times a year" (4 or more times). Collected since 2010.	Ordinal
Count of contraceptive methods on the national essential medicines list	The number of contraceptive methods included on a country's national essential medicines list (NEML). Ranges from 0-10 as defined by the World Health Organization's (WHO)'s Model Essential Medicines List,[1] plus the contraceptive patch and fertility-awareness based methods. Collected since 2010.	Continuous
Method mix strategy (private sector)	The number of FP methods (0-13) that are offered through the public, private, non-governmental (NGO) and social marketing sectors. A product is considered "offered" even if not currently in stock, if ordered or distributed within the previous 12 months, and/or that is required to be offered according to national guidelines, protocols, and laws. Collected since 2010.	Continuous
Method mix strategy (public sector)		
Fees charged to clients for FP commodities in the public sector	Assesses whether clients at public health facilities are charged fees for FP/RH commodities by policy. Collected since 2010.	Binary
Average annual SDP stockout severity greater than or equal to 25%	Average annual stockout rate of all contraceptive methods expected to be offered in the public sector at the service delivery point (SDP) level, greater than or equal to a threshold of 25%. The stockout rate for a method is calculated as the number of SDPs reported as stocked out (per the ending balance of logistics reports), summed across all reporting periods during the most recent year, divided by the total number of facilities that reported. Collected since 2010.	Binary

[1] World Health Organization Model List of Essential Medicines, 22nd List, 2021. Geneva: World Health Organization, 2021.

Supplement to: Cohen MA, Gold S, Ostrega A, Zingbagba M. National policy influences of contraceptive prevalence and method mix strategy: a longitudinal analysis of 59 low- and middle-income countries, 2010–2021. *Glob Health Sci Pract*. 2024;12(2):e2300352. https://doi.org/10.9745/GHSP-D-23-00352

**Policy/Practice**	**Definition**	**Variable Type**
Family planning charges covered by health insurance	Whether a public, government, or other national health insurance scheme covers the cost of FP services or commodities for public-sector clients (not applicable if no fees are charged). Collected since 2017.	Binary
Supplier delivery points of government- procured contraceptives at the intermediate level	Whether any government-financed contraceptives are delivered by suppliers to sub-national delivery points (not exclusive of other delivery points) below the central level and above the SDP level. Collected since 2019.	Binary
School enrollment, primary and secondary (gross), gender parity index (GPI) (ratio)	Ratio of girls to boys enrolled at primary and secondary levels in public and private schools as reported by UNESCO Institute for Statistics.	Ratio
GDP per capita (current US$)	Gross domestic product divided by midyear population. GDP is the sum of gross value added by all resident producers in the economy plus any product taxes and minus any subsidies not included in the value of the products. It is calculated without making deductions for depreciation of fabricated assets or for depletion and degradation of natural resources. Data are in current U.S. dollars. World Bank and OECD.	Monetary
CS committee has formal legal or administrative status	Whether there is a documented legislative act or ministerial decree that creates the coordination mechanism and outlines its mandate. This official process can help to clearly define how the committee operates within the Ministry of Health as well as its decision-making structures. Collected since 2010, with the exception of 2017.	Binary
CS committee policy adherence	Whether the committee has developed and implemented policies, procedures, recommendations, and/or action plans in the previous year. This indicator assesses whether the committee is engaged and active. Collected since 2017.	Binary
Existence of a government budget line for contraceptives	Whether there is a specific line in the government's budget dedicated to the procurement of contraceptives. This question does not assess whether funds have been allocated for or spent on contraceptives in any given time period. Collected since 2010.	Binary
Total expenditures as a percentage of forecast	The extent to which funds spent on contraceptives by all sources in (government and donors) the most recent 12-month period meets the amount that was forecast for that same period for the public health sector. Calculated as total government plus donor expenditures (measured by the dollar value of contraceptives delivered during the most recent completed 12-month period) divided by the dollar value of the contraceptive forecast for the same period. Collected since 2010.	Percentage
Existence of a CS strategy	Whether there is a current national strategy in place that includes objectives for contraceptive security. This indicator demonstrates whether national policy acknowledges and supports CS and has a strategy in place to manage and work toward it. Collected since 2010.	Binary

Supplement to: Cohen MA, Gold S, Ostrega A, Zingbagba M. National policy influences of contraceptive prevalence and method mix strategy: a longitudinal analysis of 59 low- and middle-income countries, 2010–2021. *Glob Health Sci Pract*. 2024;12(2):e2300352. https://doi.org/10.9745/GHSP-D-23-00352

**Policy/Practice**	**Definition**	**Variable Type**
Implementation of a CS strategy	Whether there is evidence of implementation of action items that are part of the contraceptive security strategy, and/or follow up on addressing issues raised in the strategy. Responses of 'high level of implementation' and 'some implementation' in 2019 and 2021 are combined to indicate implementation, while 'minimal or no implementation' is considered no implementation.<br>Collected since 2010.	Binary
FP commodities subject to duties - all sectors	Whether FP commodities procured by at least one of four sectors (public, private, NGO, and social marketing) are subject to import duties. Collected since 2019.	Binary
FP commodities subject to duties - public sector	Whether FP commodities procured by the public sector are subject to import duties. Collected since 2010.	Binary
Fees charged to clients for FP services in the public sector	Assesses whether clients at public health facilities are charged fees for FP/RH services by policy. Collected since 2010.	Binary
Promotion of FP by channel – social marketing	Assesses whether family planning is actively promoted through the channels of social marketing, mass media, and community mobilization and engagement. Promotion of FP may include highlighting the benefits, familiarizing people with the services and commodities available, where to access them and at what cost, explaining the different methods, addressing any concerns about product safety and side effects, and/or dispelling misconceptions. These activities may be implemented by any sector or combination of sectors. The responses were scaled as follows: 'no promotion'=0, 'some promotion'=1, and 'extensive promotion'=2.<br>Collected since 2019.	Ordinal
Promotion of FP by channel – mass media		Ordinal
Promotion of FP by channel – community mobilization and engagement		Ordinal
Approximate percentage range of FP providers trained in implant and IUD insertion and removal	The estimated percentage range, in increments of 20 percentage points of family planning service providers in the public sector who have received training on implant and IUD insertion and removal, according to national surveys, human resources systems, and other government records.<br>These values were transformed into a scale as follows:<br>0 = (0 -10%) + (11-20%)<br>1 = (21-30%) + (31-40%)<br>2 = (41-50%) + (51-60%)<br>3 = (61-70%) + (71-80%)<br>4 = (81-100%)<br>Collected since 2019.	Ordinal
Global Financing Facility partnership in place	Whether the country has a formal partnership in place with the Global Financing Facility. Collected since 2017.	Binary

Supplement to: Cohen MA, Gold S, Ostrega A, Zingbagba M. National policy influences of contraceptive prevalence and method mix strategy: a longitudinal analysis of 59 low- and middle-income countries, 2010–2021. *Glob Health Sci Pract*. 2024;12(2):e2300352. https://doi.org/10.9745/GHSP-D-23-00352

**Policy/Practice**	**Definition**	**Variable Type**
Supplier delivery points of government- procured contraceptives at the central level	Whether any government-financed contraceptives are delivered by suppliers to a central-level delivery point (not exclusive of other delivery points). Collected since 2019.	Binary
Supplier delivery points of government- procured contraceptives at the SDP level	Whether any government-financed contraceptives are delivered by suppliers directly to SDPs (not exclusive of other delivery points). Collected since 2019.	Binary
Government procures contraceptives at the central level	Whether any government-financed contraceptives are procured from the central level (not exclusive of other procurements that take place at other administrative levels). Collected since 2019.	Binary
Government procures contraceptives at the intermediate level	Whether any government-financed contraceptives are procured from the sub-national administrative level, which is below the central level and above the SDP level (not exclusive of other procurements that take place at other administrative levels). Collected since 2019.	Binary
Government procures contraceptives at the SDP level	Whether any government-financed contraceptives are procured from the SDP level (not exclusive of other procurements that take place at other administrative levels). Collected since 2019.	Binary
Average annual central stockout severity greater than or equal to 25%	Average annual stockout rate of all contraceptive methods expected to be offered in the public sector at the central level, greater than or equal to a threshold of 25%. The stockout rate for a method is calculated as the number of central level stock status observations of a stockout, summed across all reporting periods during the most recent year, divided by the total number of central level stock status observations in the same period. Collected since 2010.	Binary
